# Biological Properties, Bioactive Constituents, and Pharmacokinetics of Some *Capsicum* spp. and Capsaicinoids

**DOI:** 10.3390/ijms21155179

**Published:** 2020-07-22

**Authors:** Gaber El-Saber Batiha, Ali Alqahtani, Oluwafemi Adeleke Ojo, Hazem M. Shaheen, Lamiaa Wasef, Mahmoud Elzeiny, Mahmoud Ismail, Mahmoud Shalaby, Toshihiro Murata, Adrian Zaragoza-Bastida, Nallely Rivero-Perez, Amany Magdy Beshbishy, Keneth Iceland Kasozi, Philippe Jeandet, Helal F. Hetta

**Affiliations:** 1 Department of Pharmacology and Therapeutics, Faculty of Veterinary Medicine, Damanhour University, Damanhour 22511, AlBeheira, Egypt; dr_hazemshaheen3010@yahoo.com (H.M.S.); lamiaawasef@vetmed.dmu.edu.eg (L.W.); mahmoudelzeiny755@gmail.com (M.E.); mahmoudesmail7899@gmail.com (M.I.); eidm98282@gmail.com (M.S.); 2 Department of Pharmacology, College of Pharmacy, King Khalid University, Guraiger, Abha 62529, Saudi Arabia; amsfr@kku.edu.sa; 3 Department of Biochemistry, Landmark University, Omu-Aran 251101, Nigeria; ojo.adeleke@lmu.edu.ng; 4 Department of Pharmacognosy, Tohoku Medical and Pharmaceutical University, Aoba-ku, Sendai 981-8558, Japan; murata-t@tohoku-mpu.ac.jp; 5 Área Académica de Medicina Veterinaria y Zootecnia, Instituto de Ciencias Agropecuarias, Universidad Autónoma del Estado de Hidalgo, Rancho Universitario Av. Universidad km 1, EX-Hda de Aquetzalpa, Tulancingo, Hidalgo 43600, Mexico; adrian_zaragoza@uaeh.edu.mx (A.Z.-B.); nallely_rivero@uaeh.edu.mx (N.R.-P.); 6 National Research Center for Protozoan Diseases, Obihiro University of Agriculture and Veterinary Medicine, Nishi 2-13, Inada-cho, Obihiro 080-8555, Hokkaido, Japan; 7 Infection Medicine, Deanery of Biomedical Sciences, College of Medicine and Veterinary Medicine, The University of Edinburgh, 1 George Square, Edinburgh EH8 9JZ, UK; kicelandy@gmail.com; 8 Research Unit “Induced Resistance and Plant Bioprotection”, EA 4707, SFR Condorcet FR CNRS 3417, Faculty of Sciences, University of Reims, PO Box 1039, CEDEX 2, 51687 Reims, France; philippe.jeandet@univ-reims.fr; 9 Department of Medical Microbiology and Immunology, Faculty of Medicine, Assiut University, Assiut 71515, Egypt; 10 Department of Internal Medicine, University of Cincinnati College of Medicine, Clifton Ave, Cincinnati, OH 45221, USA

**Keywords:** bioactive constituents, *Capsicum* spp., pharmacokinetics

## Abstract

Pepper originated from the *Capsicum* genus, which is recognized as one of the most predominant and globally distributed genera of the Solanaceae family. It is a diverse genus, consisting of more than 31 different species including five domesticated species, *Capsicum baccatum*, *C. annuum*, *C. pubescen*, *C. frutescens*, and *C. chinense.* Pepper is the most widely used spice in the world and is highly valued due to its pungency and unique flavor. Pepper is a good source of provitamin A; vitamins E and C; carotenoids; and phenolic compounds such as capsaicinoids, luteolin, and quercetin. All of these compounds are associated with their antioxidant as well as other biological activities. Interestingly, *Capsicum* fruits have been used as food additives in the treatment of toothache, parasitic infections, coughs, wound healing, sore throat, and rheumatism. Moreover, it possesses antimicrobial, antiseptic, anticancer, counterirritant, appetite stimulator, antioxidant, and immunomodulator activities. Capsaicin and *Capsicum* creams are accessible in numerous ways and have been utilized in HIV-linked neuropathy and intractable pain.

## 1. Introduction

Chili peppers originated after the Columbian Exchange in Mexico; various types of chili pepper spread through the planet, employed in the diet and as a traditional medicine [1]. Pepper (*Capsicum* spp.) is among the oldest cultivated and most employed crops. Its usage started quite a long time ago and it is considered to have its source in America [2,3]. The genus *Capsicum* contains over 200 species, with the fruits differing extensively in taste and olfactory heat. Five principal *Capsicum* types mentioned in published works include: *Capsicum annuum*, *C. baccatum*, *C. chinense*, *C. frutescens*, and *C. pubescens*. Peppers from *Capsicum* species are natural to the tropical and humid zones of Central and South America and incorporate peppers of significant financial importance [4,5]. They are generally employed as condiments or nutrients, with a wide range of beneficial use in Indian, American, and Chinese therapeutic customs for the treatment of acne, inflammation of the joints, and indigestion [6]. Pepper belongs to the genus *Capsicum*, which is an associate of the Solanaceae family. The genus *Capsicum* contains roughly 31 types, of which the major five cultivated types are *C. annuum*, *C. baccatum*, *C. chinense*, *C. frutescens*, and *C. pubescens. C. frutescens* and *C. chinense* are now widely cultivated [3]. Pepper is the most generally utilized seasoning sauce globally and is markedly valued for its spiciness and adds exceptional savor to numerous dishes all over the world. In the past, it was utilized primarily for flavorings and as a therapeutic plant, but nowadays its usage has expanded to crisps, prepared vegetables, and flavorings; it is reared as a decorative plant; and it is used in the preparation of extracts for numerous medicinal and make-up businesses [7,8]. The normal weekly family intake of dry pepper is projected at 140 g. It is taken as fine particles processed from dry pods called “*berbere*” and mixed with dishes as a food and flavor. The pods are the major constituents for processing “*shiro*” powder, recognized as a common condiment [9]. Herbal medicine remains generally exercised in major emerging countries, whereas the process of employing alternate medicine is rapidly cumulative [10,11]. In Eritrea, the usage of herbal medicine is comparable to several countries with copious therapeutic traditions in numerous situations [12,13]. Pepper is a major plant employed as medication over time in several countries. In ancient times, it was employed for managing respiratory disease and alleviating toothaches [3]. In accordance with the World Health Organization, inquiries about its toxicity, safety, potency, value, accessibility, protection, and additional improvement are required. It was revealed that about 4 out of 10 people in the UK employ alternative medicine sometimes in their lives [14]. Therefore, the aim of this work is to focus on the nutritional value, morphology and extraction procedures as well as the therapeutic properties and uses, recommended doses, toxicity, and pharmacokinetics of chili pepper.

## 2. Morphology and Extraction Procedures

*C. annuum*, which is a suffrutescent annual shrub, grows up to 0.75–1.8 m in cultivated locations with many angular twigs. The leaves are simple and are of different shapes, and alternate, elliptical to lanceolate, with smooth margins (entire) that are usually wrinkled. The small flowers (around 1.5 or 1 cm in diameter) are white or violet, in groups of two or more. The fruits are many-seeded berries which may be long, cylindrical, ovoid, obtuse, or oblong, but with no sutures; they are red when ripe, with a smooth shiny surface. The fruit is up to 25 cm in length and 7 mm in breadth, with many seeds which are yellow, smooth, round, and discoid, with a protuberant spine-aroma on the edge. *C. annuum* fruits have a characteristic odor and pungent taste [15]. Several extraction procedures of capsaicinoids from hot peppers have been established in the past few years. When planning an extraction procedure, the initial step is the choice of a suitable solvent that would give a good yield of the preferred compound. Amongst the solvents employed for extracting capsaicinoids, methanol, ethanol, acetonitrile, and water are the most common solvents [16]. Furthermore, in the solvent choice process, numerous other prompting factors are deliberated to attain a high extraction productivity—for example, the temperature, time of extraction, solvent volume, sample quantity, and the replicability and reproducibility of the procedures. The methods that are extensively used by researchers include maceration [17], magnetic stirring [18], enzymatic extraction [19], microwave- [20] and ultrasound-assisted extraction (UAE) [21], Soxhlet (SOX) [22], supercritical fluid [23], and pressured liquids extraction (PLE) [24]. The general extraction procedures for capsaicinoids are discussed in the following.

### 2.1. Enzymatic Treatment

Enzymatic approaches are suggested to increase the yield of compounds extracted from fruits [25]. The study conducted by Santamaria et al. [19] reported that a number of commercially available enzymes were used to separate chili pepper tissues and increase yields by 7%, with a final recovery of 80% of capsaicinoids. Recently, a comparable treatment protocol was espoused by Desikacharya et al. [26] via extrazyme (mainly pectinase and multiple carbohydrases) and energex (mainly glucanase), which augmented the capsaicinoid yield by 32%. In accordance with the treatment protocols specified above, Salgado-Roman et al. [27] projected a nonsalable enzymatic action via the enzymatic extracts obtained from *Rhizopus nigricans*.

### 2.2. UAE (Ultrasound-Assisted Extraction)

The UAE method is productive owing to the occurrence of cavitation, which happens once an ultrasonic wave transients via an organic solvent, generating energy to improve the mixing and diffusion of the solvent into the sample matrix [21]. The functions of UAE offer numerous merits, for example the reduction in the solvents, temperature, and time needed for extraction, which is vital for the extraction of thermolabile and unstable compounds [28] Barbero et al. [18] established a fast-replicated UAE protocol for capsaicinoids from three types of peppers.

### 2.3. Soxhlet (SOX) Extraction

The SOX process is an orthodox technique that is extensively helpful in removing oil from the matrix, which is utilized when the compound of interest has restricted solubility in a solvent while the impurities are insoluble in the solvent [22]. Bajer et al. [29] extracted capsaicinoids from several chili samples via the SOX procedure, with methanol as the solvent and an extraction time of 2 h. The same SOX procedure was utilized in a study by Liu et al. [30], in which the extraction of 1.0 g of a *C. annuum* sample was achieved with 50 mL of methanol for 2 h.

### 2.4. Supercritical Fluid Extraction (SFE)

Supercritical fluids are constituents at pressures and temperatures above their critical values and are powerful solvents for nonpolar compounds [31]. After the pressure is attuned to ambient pressure, the supercritical fluids return to the gas phase and vaporize without leaving solvent residues. Supercritical fluid extraction (SFE) is utilized as an alternate to the orthodox procedure during the extraction of bioactive compounds, with the benefit of moderate temperatures, decreased energy consumptions, and high-purity extracts [32]. Carbon dioxide (CO_2_) is regularly utilized as the supercritical solvent for capsaicinoids because it is cheap, nontoxic, nonflammable, and inert and has a high extraction capacity [23,33,34]. Capsaicinoid extraction from the malagueta pepper (*C. frutescens* L.) can be performed via SFE aided by ultrasound with CO_2_ as the solvent at the pressure, temperature, and flow rate of 15 MPa, 40 °C, and 1.673 × 10^−4^ kg/s, respectively [35]. The improved SFE rate was attained when the ultrasound power was maintained at 360 watts for 60 min. Later in 2016, Dias et al. [32] accomplished a similar SFE test on dedo de moça pepper with 25 MPa, 40 °C, 600 W, and 80 min and without 25 MPa, 40 °C. The results revealed that the universal yield of SFE was productively improved.

### 2.5. PLE (Pressurized Liquids Extraction)

The process of PLE is regularly performed at a raised temperature and pressure, allowing the high solubility of the compound in the solvent whilst maintaining the solvent beneath its boiling point, and thus leading to a high permeation of the solvent into the sample matrix [24,36]. Many scientists have espoused the PLE technique [29,30,37]. Barbero et al. [37] improved a PLE protocol with the extraction solvent of water, methanol, and ethanol with a temperature of 200 °C and pressure of 100 atm.

### 2.6. MAE (Microwave-Assisted Extraction)

The method of microwave-assisted extraction (MAE) is performed via the mixture of microwaves and a conventional solvent that employs energy production in numerous experiments [20,38,39]. In agreement with Williams et al. [20], the capsaicinoids yielded via the MAE technique multiplied and the time was considerably reduced when associated with the conventional reflux and jarring flask extraction procedures. The MAE requirements for the extraction of capsaicinoids from fresh pepper samples were enhanced by Barbero et al. [38]. The authors also compared it to the extraction effectiveness of generally utilized protocols—for example, magnetic stirring—and established that MAE is a much quicker technique. Chuichulcherm et al. [39] made an evaluation of three diverse extraction procedures (SOX, MAE, and UAE).

## 3. Chemical Constituents

### 3.1. Capsicum Annuum

The chemical composition of *C. annuum* fruits include dry matter, total fat, protein, carbohydrates, dietary fibers, vitamin C, calories, and energy [40]. Zaki et al. [40] determined the nutritional and biochemical constituents of *C. annuum* fine particles (dry weight, DW) at diverse harvest periods (December, November, October, and September), as shown in Table 1. However, the mineral constituents of the oils extracted from two varieties of *C. annuum* (sweet and bell pepper) are shown in Table 2 [41].

The fruits of *C. annuum* contained capsaicinoids, a kind of bioactive compound that provide a distinctive sharp taste. Two main capsaicinoids, capsaicin and dihydrocapsaicin, were accountable for 90% of the total sharp strong taste of pepper fruits. In addition to capsaicin and dihydrocapsaicin, at least nine insignificant capsaicinoids have been revealed to appear in peppers [42]. Capsaicin (C_18_H_27_NO_3_) is identified with numerous alternative expressions—i.e., *N*-[(4-hydroxy-3-methoxybenzyl]-8-methyl-*trans*-6-nonenamide, *N*-[(4- hydroxy-3-methoxy-phenyl)methyl]-8-methyl-*trans*-6-nonenamide, *N*-(3-methoxy-4-hydroxy benzyl)-8-methylnon- *trans*-6-enamide, *trans*-8-methyl-*N*-vanillyl-6- nonenamide, isodecenoic acid vanillylamide, and 8-methylnon-6-enoyl-4-hydroxy-3-methoxy benzylamide. Capsaicin is a decylenic acid amide of vanillyl-amine. On the alteration in the acid part of the particle, a dissimilar grade of sharpness of analogues has been perceived [43,44]. Capsaicin is an odorless white crystal with an intense sharpness. One part in 100,000 can be sensed by tasting. It has a molecular weight of 305.4118 g/mol, a melting point of 65 °C, a boiling point at 0.01 mm Hg of 210–220 °C, a sublimate at 115 °C, a UV max at 227 and 281 nm, and is faintly soluble in carbon disulfide and hot water [45]. The capsaicin level of the fruits is principal in the burning taste of several kinds of *Capsicum* sp. The capsaicin content was determined in 12 types of edible *capsicum* in different areas in Indonesia. The results indicated that green paprika, yellow paprika, and red paprika do not contain capsaicin [46]. The chemical constituents of *n*-hexane extracts from *C. annuum* at diverse phases of maturing (red, green, and small green) are shown in Table 3 [47]. Nine compounds were observed in the flavonoid and phenolic acid fraction, as shown in Table 4.

The key compounds of this fraction obtained from red pepper were sinapoyl and feruloyl glycosides, and the major compound from green pepper was quercetin-3-*O*-l-rhamnoside [50]. The leaves contain alkaloids, tannins, and flavonoids [51], whereas the roots contain steroids, alkaloids, coumarins, glycosides, and triterpenoids [52]. Ten sesquiterpenoids were isolated from the ethyl acetate soluble fraction of the methanolic extract of the dried stems and roots of *C. annuum* [53]. *C. annuum* contained a considerable quantity of L-asparaginase. The enzyme purified from the plant enzyme existed in two ways, only one with antitumor activity. The purified enzyme has a molecular weight of 120,000 ± 500. The *N*-terminal and the *C*-terminal amino acids residues are alanine and phenylalanine. The enzyme has indivisible glutaminase activity and urease activity [54].

### 3.2. Capsicum Frutescens

Many bioactive compounds were isolated from *C. frutescens*, including essential oils, alkaloids, glycosides, phenolic compounds, flavonoids, esters, terpenoids, noncarotenoids, lipoxygenase derivatives, carbonyls, alcohols, hydrocarbons, hydroxybenzoic acid, hydroxycinnamic acid, ascorbic acid, tannins, steroids, capsaicin, dihydrocapsaicin, capsiconinoids, capsinoids, *ortho*-hydroxy-*N*-benzyl-16- methyl-11,14-diene-octadecamide, and 9 and 12-diene-octadecanoic acid. Capsinoids are non-pungent capsaicin-related compounds derived from the CH-19 sweet pepper of all varieties of the *Capsicum* genus. Capsiate, dihydrocapsiate, and nordihydrocapsiate are three capsinoids structurally similar to the pungent capsaicins, dihydrocapsaicin and nordihydrocapsaicin, which are, respectively, the bioactive constituents of *Capsicum*, except for the fact that their two moieties are connected by an ester bond instead of an amide bond [55]. However, the capsaicinoids (vanillylamides of monocarboxyl acids) which are accountable for the sharpness are believed to be the bioactive compounds in the plants’ fruits [56,57].

## 4. Evaluation of Biochemical Composition

*C. annuum* fruits are rich in capsaicinoids, carotenoids, flavonoids, vitamins, and minerals (Table 5). Studies conducted in the last 50 years have contributed in discovering the type and the percentage of the main chemical constituents in the plants of this species. The composition and quantity of these compounds vary according to the genotype; moreover, other factors—e.g., the fruit ripening and the cultivation system—could influence the composition. From a genetic perspective, the occurrence/nonappearance of capsaicinoids varies in the fruit placenta, while the alternative presence of three recessive alleles (*pun1*, *pun1^2^*, *pun1^3^*) determine the absence of these compounds. These metabolites could be a natural defense against biotic and abiotic mechanisms [58]. Furthermore, the presence of other phytochemical substances is favorable for the plant and for the human being; for example, the antioxidant activities of polyphenols, such as flavonoids and cinnamic acid derivates, could be a gastric protector in the human and animal diet. Capsaicin (also called 8-metil-*N*-vanillil-6-noneamide, C_16_H_27_NO_3_) and diidrocapsaicin are responsible for 90% of the spice [59]. Both, together with other minor capsaicinoids, such as omodiidrocapsaicin, nordiidrocapsaicin, and omocapsaicin, are the major end products of the metabolic pathway that allows the synthesis of these compounds throughout vanillylamide condensation with branched short chain fatty acids. The capsaicin is the most powerful natural compound, with a pungent, painful, and desensitizing effect, since its structure has an ammidic bond and a double bond (linked to an ether). Variations in one or more groups (chain length, branches, etc.) indicate the intensity of the desensitization. Capsaicinoids, which are synthesized in the fruit placenta after enzymatic condensation, are alkaloid, which confers to the fruits a strong and pungent taste that is popular as a spice; they are characterized by the presence of a nitrogen atom. This atom is not part of a heterocyclic ring, like the other alkaloid alkaloids; for this reason, the capsaicinoids are classified as proto-alkaloids or pseudo-alkaloids [60]. As the fruits ripen, the capsaicinoids start to increase. The seeds do not yield capsaicin, but they can recover it from tissues of the plant that surround them. Capsaicin and other capsaicinoids are stable alkaloids—they stay intact also after cooking and thawing for a long period. Capsaicinoids and capsaicin produce a stinging feeling in mammals, including humans, and produce a burning sensation in the mucosa and the mouth, where they stimulate the VR1 receptors (Vanilloid Receptor type 1). These receptors activate the VRL-1 (Vanilloid receptor-like) protein synthase, the enzyme which produces capsaicinoids and catalyzes the reactions that produce two metabolites: vanilalanine (a biosynthetic type of phenilpropanoid) and 8-methyl-6-nonenone (a biosynthetic type of valine). Activated capsaicin connects specific membrane receptors (vanilloid receptor) located on primary afferent nerves. They cause a flow of calcium ions, which depolarize nervous fibres and send nervous pulses to the brain, provoking pain. After the initial excitation, which provokes the burning sensation, during a period of analgesia the neuron cannot answer to any nociceptive stimulus. Secondary metabolites of the phenolic group are synthetized from the plant during stress conditions. Piperine is an *N*-acylpiperidine that belongs to the vanilloid family of compounds, including capsaicin, with an antimutagenic activity. Recently, they have been studied by researchers, as they are antioxidants and can protect organisms from free radicals produced during the cell metabolism. Several studies have tried to evaluate the presence and the dose of phenols in plants of the *Capsicum* genus. Among them, Sukrasno et al. [61] highlighted the presence of two flavonoids—3-*O*-ramnosilquercetin and 7-*O*-glucosilluteolin—and three acid cinnamic derivatives: glucoside *p*-cumaroyl-cinnamoyl, glucoside caffeoyl-cinnamoyl, and 3,4-dimethoxycinnamoyl glycoside. Other researchers have evaluated the composition of two flavonoids (quercetin and luteolin) after the acid hydrolysis of a phenolic portion of a *C. annuum* extract [62]. Loizzo et al. [63] achieved a detailed analysis of a phenolic chili pepper portion and identified 10 compounds. Three of them were new: capsiosid A, capsiosid B, and capsianosid VII. Other phenols in plants of the *Capsicum* genus have been reported, including *trans*-*p*-feruloyl-β-d-glucopyranoside, *trans*-*p*-sinapoyl d-glucopyranoside, apigenin 6-*C*-d-glucopiranoside-8-*C*-l-arabinopiranoside, d-glucopyranoside, quercetin 3-*O*-l-ramnopyranoside-7-*O*-d-glucopyranoside, quercetin 3-*O*-l-ramnopiranoside, luteolin 7-*O*-[2-d-apiofuranosyl)-4-d-glucopyranosyl)-6-malonyl-d-glucopyranoside, luteolin 6-*O*-d-glucopyranosyl-8-*C*-l-arabinopyranoside, and luteolin-7-*O*-[2-d-(apiofuranosyl)-β-d-glucopyranoside] [59]. Flavonoids are the other compound with an antioxidant property. They stop the activity of some enzymes, such as prostaglandin synthase, lipoxygenase, and cyclooxygenase, which are involved in cancer genesis. In fruits and plants, they are glycosides, with a sugar linked to 3-carbon. They are degraded to aglycones after ingestion. Quercetin and luteolin are the principal polyphenolic flavonoids found in *C. annuum*. Carotenoids are responsible for the product color, and they are at epicarp. The coloring is the result of more than 30 types of carotenoids: capsanthin and capsorubin are accountable for the red color, while the yellow shade is given by xanthophylls and carotenes. All the carotenoids in chili peppers are isoprenoids with 40 carbon atoms and contain nine double bonds in the central chain; the diverse final groups (b, e, k, 3-idrossi-5, 6-eposide) contain the modification chromophore properties of all pigments, permitting their classification into two isochromatic families: red (R) and yellow (Y). The red portion is rich in capsanthin, capsanthin-5,6-episode capsorubin (together with other minor carotenoids), while the yellow portion consists of all the other pigments (principally zeaxanthin, violaxanthin, anteraxanthin, β-cryptoxanthin, β-carotene, cucurbitaxanthin A). β- Carotene is a naturally occurring retinol (vitamin A) precursor obtained from certain fruits and vegetables with potential antineoplastic and chemopreventive activities. Peppers are also a rich supply of oxygenate carotenoids, which differ in components and concentration according to their genetics and ripening grade [64]. From dry chili peppers, it is possible to extract an oleoresin, which is used as a food dye. Peppers are also riches in vitamins [65]. Vitamins are essential organic compounds. They must be taken daily with the diet in small amounts, as they are not synthetized by the organism. In the *Capsicum* genus, the plants are abundant in vitamins A, C, E, D, and B [66]. Ascorbic acid has antioxidant properties in biologic organisms and restricts worsening processes [67]. Ascorbic acid is the primary molecule that is biologically active, but deidroascorbic acid is important for humans because it is easily transformed into ascorbic acid.

## 5. Pharmacological Activity

### 5.1. Traditional Uses

**Figure 1:**
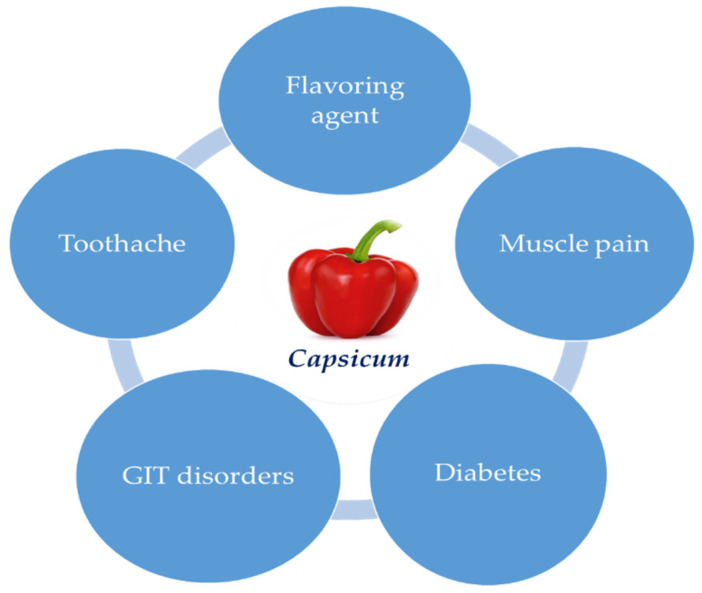
Traditional uses of *Capsicum*.

*Capsicum* is a humid, significant agrarian crop and one of the most prevalent vegetables, not only due to its financial worth, but for the mixture of the color, taste, and nutritious properties of its fruit [75,76] (Figure 1). The interest in the ingesting of *Capsicum* is, to a great degree, because of its constituents and significance as a dietary antiradical [4].

*Capsicum* has been utilized as a colorant, flavorant, and/or as a source of spiciness. The key supply of sharpness in peppers is the functional group of alkaloid components, known as capsaicinoids (CAPS), formed in the fruit. Dried chili is likewise appreciated for its involvement in the taste of chili sauces and chili powders. Its flavoring property is linked to its volatile aromatic compounds and color. As a universal rule, when the color of paprika or chili powder disappears, the flavor also vanishes [4]. Both volatile and non-volatile substances add to its usage as a flavoring agent [77]. *C. annuum* was used traditionally to cure toothache. The fruits are employed to activate gastric activities and cause an upsurge in blood circulation. It is also a stimulant and a carminative, and is utilized traditionally for neuralgia and rheumatism [78,79]. *C. frutescens* was also utilized locally as an exterior remedy in agonizing muscle pangs. Furthermore, it is also employed for managing hyperglycemia, blood pressure (high/low), bronchitis, and burning feet; to upsurge circulation; to ease rheumatic discomfort; to heal mouth wounds and infested wounds; to decrease blood clots; and to aid ingestion by accelerating the saliva and stomach juice flow [80,81]. Capsaicin and its derivatives were employed topically to cure chronic discomfort syndromes, musculoskeletal pain, and diabetic complications [82,83]. The topical usage of capsaicin induces burning pain and neurogenic inflammation [84,85]. The cured area becomes less sensitive to pain after recurrent applications; this outcome has made capsaicin a slightly acting analgesic in chronic painful complains [86,87].

### 5.2. Activities Related to Infectious Diseases

#### 5.2.1. Antimicrobial Effects

##### Capsicum annuum

The butanol extract of *C. annuum* fruit revealed a great antimicrobial activity compared to verified pathogens, though other extracts have displayed a relatively modest activity. The ethanol extract (100 mg/mL) of *C. annuum* has displayed a relatively great antimicrobial activity against *Micrococcus sp* (20 mm)*, Bacillus* (10 mm), *E. Coli* (17 mm)*, Pseudomonas* sp (16 mm), and *Citrobacter* sp (15 mm) [88]. The inhibitory effect of the extract of *C. annuum* bell pepper type was examined against *Salmonella typhimurium* and *Pseudomonas aeruginosa*, injected in pulverized beef meat mixed with diverse concentrations of the extract, and stored at 7 °C for 7 days [89]. The antibacterial activity of *C. annuum* was examined against pathogenic strains acquired from the urinary tract (*Klebsiella pneumoniae*, *Pseudomonas aeruginosa*, and *E.coli*). The diverse concentrations of the plant extracts displayed an antibacterial activity at 5 and 10 mg/mL against the tried microorganisms [90].

##### Capsicum frutescens

*C. frutescens* exhibited antibacterial and antifungal activities. CAY-1, a novel saponin obtained from *C. frutescens,* was identified to be potent against 16 diverse fungal strains; its mechanism of action is by rupturing the membrane integrity of fungal cells [88,91]. The minimum inhibitory concentration (MIC) of *C. frutescens* was estimated against six strains of Gram-positive (*Staphylococcus aureus, Enterococcus faecalis*, *Bacillus subtilis*) and Gram-negative (*Escherichia coli*, *Klebsiella pneumonia*, *Pseudomonas aeruginosa*) bacteria and one yeast strain (*Candida albicans*), but for all of these microorganisms, the required concentrations were greater than 1000 μg/mL [92].

#### 5.2.2. Antifungal Effect

The antifungal properties of *C. frutescens* extracts against four main fungal strains related to groundnut storage (*Aspergillus flavus, A. niger, Penicillium* sp., and *Rhizopus* sp.) were examined. The MIC and minimum fungicidal concentration (MFC) of *C. frutescens* extracts were evaluated. The MIC values of the fruit extract decreased compared to the leaf extract. At the MIC, the leaf extract revealed a potent activity against *A. flavus* (88.06%), while the fruit extract revealed activity against *A. niger* (88.33%) in the well diffusion method. Groundnut seeds treated with *C. frutescens* fruit extract (10 mg/mL) displayed an advanced rate of fungal inhibition [93]. Moreover, the peptides obtained from chili pepper seeds hindered the development of the yeasts *Saccharomyces cerevisiae, Candida albicans, C. parapsilosis, C. tropicalis, Pichia membranifaciens*, *Kluyveromyces marxiannus*, and *C. guilliermondii*. The peptide fraction displayed a potent fungicidal activity against *C. albicans, Saccharomyces cerevisiae*, and *Schizosaccharomyces pombe*, and also fostered numerous morphological differences to *C. albicans*. It also decreased the glucose-activated acidification of the medium facilitated by the H (+)-ATPase of *S. cerevisiae* cells in a concentration-dependent approach and resulted in the permeabilization of the yeast membrane to the dye SYTOX Green, as established by confocal laser microscopy [94].

#### 5.2.3. Antiviral Effect

Hafiz et al. [95] documented the antiviral effects of *C. annuum* crude extracts against herpes simplex virus-1 (HSV-1) and HSV-2 with a low cytotoxic activity. They revealed that this activity was attributed to the presence of several bioactive compounds, including ascorbic acid and ß-carotene.

#### 5.2.4. Insecticidal, Anthelmintic and Larvicidal Effects

The insecticidal activities of red pepper (*C. annuum* L.) fruit powder were examined against *Rhyzopertha dominica* and *Sitophilus granaries*. The results revealed that red pepper in low concentrations did not cause complete mortality in two insects after 14 days. It caused a complete reduction in the Fl progeny of *S. granarius* and *R. dominica* at the highest tested dosages [96]. *C. annuum* and *C. frutescens* fruit and seed powders were studied in the research center for the regulation of *Callosobruchus maculatus* (F.) in stowed cowpea and *Sitophilus zeamais* in stowed maize. *C. frutescens* and *C. annuum* seed powder dust were poisonous to *C. maculatus* and *S. zeamais* at the rate of 5.0 g and 7.5 g per 50 g of cowpea and of 50 g maize within 48 h and 96 h, respectively [97]. The mosquito-repellent effect of *C. frutescens* extracts was studied against *A. aegypti* mosquitoes. The plant parts were air-dried in the shade and crushed to powder. The combination of *C. frutescens* and *C. papaya* was efficient for 4 h, while that of *C. frutescens* and *C. dactylon* was efficient for 3 h. The combination of all three extracts was efficient for 4 h, i.e., allotting the same period of defense as the combination of *C. frutescens and C. papaya*. Combinations of extremely repellent extracts are likely to produce high-repelling products, though it is not possible for the repellency of the mixture to be a simple additive product of the repellencies of the constituent extracts [98]. The insecticidal activity of distinct concentrations of the methanol extract of the fruits and leaves of *C. frutescens* was investigated against the 2nd and 3rd instar larvae of *A. aegypti*. The mortality of the larvae was discovered to be dose-dependent. The fruit extract has displayed an additional killing property compared to the leaf extract. The antiparasitic effect of the aqueous extract of *C. frutescens* against the fish ectoparasite *Ichthyophthirius multifiliis* was estimated under in vitro and in vivo circumstances. The outcomes in vitro indicated that aqueous *C. frutescens* extracts led to a more than 70% mortality of *I. multifiliis* theronts during 4h of exposure and considerably decreased the existence of the tomonts and the total number of theronts freed by the tomonts within 22 hhr [99]. In an in vitro study, extracts from the leaves of *C. annuum* resulted in the death of the cercaria of *Schistosoma mansoni* within 15 min. The active principles appeared to be water-soluble unsaturated compounds from the oils or their hydrolysis products [100]. The ethanol extract of *C. annuum* has been shown to have a larvicidal activity against *Anopheles stephensi* and *Culex quinquefasciatus*. The treated larvae revealed curling up agitation and active body movements that were the characteristics of neurotoxicity.

### 5.3. Biological Activity

#### 5.3.1. Antioxidant Properties

*C. annuum, C. chinense*, and *C. frutescens* contain an extensive range of secondary metabolites with renowned antioxidant properties [101]—for example, carotenoids [102]; capsaicinoids [17,103,104]; and phenolic compounds, predominantly flavonoids, quercetin, and luteolin [74,105,106,107]. Rosa et al. [103] showed that capsiate, dihydrocapsiate, and their analogues possess a highly significant antioxidant activity. The ingestion of *C. annuum* for 4 weeks was discovered to elevate the resistance of serum lipoproteins to oxidation in adult men and women, the antioxidant property of capsaicinoids giving additional advantage in the management of cardiovascular diseases [108]. The carotenoids extracted from dried *C. annuum* were also examined for their antioxidant activities [109].

#### 5.3.2. Anti-Inflammatory Properties

*C. annuum* displayed hypocholesterolemic properties in animal assays [110,111]. A recent experiment established that hyperlipidemia, inflammation, and oxidative stress are closely associated with the pathogenesis of atherosclerosis and, subsequently, with the augmented risk of cardiovascular diseases [112,113]. Hence, an agent that has antioxidant and anti-inflammatory activities was beneficial in the deterrence of these pathologies. Chili pepper *C. baccatum* var. *pendulum* is broadly ingested in Brazil, and a few studies discovered in the literature place emphasis on its carotenoid and capsaicinoid constituents and the antioxidant activity of its crude juice [107,114,115]. Topical capsaicinoid-containing patch applications or local capsaicin injections (2, 10, 20 μg/paw) alone did not have an effect on edema volume and weight. However, the mixture of diclofenac with topical capsaicinoid-containing patches considerably upsurged the efficiency of diclofenac on inflammation [116]. The anti-inflammatory effects of the ethyl acetate extract of *C. frutescens* (CFE) were studied on rat hind paw inflammation induced by subplantar injections of fresh egg albumin (0.5 mL/kg). The ethyl acetate extract of *C. frutescens* generated anti-inflammatory effects that were similar to diclofenac [117]. In addition, *C. annuum* showed an anti-inflammatory activity by inhibiting the Soyal lipoxygenase (LOX) enzyme. The outcome displayed a greater % of LOX inhibition by green *Capsicum* (46.12%), followed by yellow (44.09%) and red *Capsicum* (32.18%) [118]. Carotenoid extracts from dried *C. annuum* displayed substantial peripheral analgesic activities at 5, 20, and 80 mg/kg and induced central analgesia at 80 mg/kg. The guajillo pepper carotenoid extracts also revealed anti-inflammatory activity; they considerably thwarted edema formation at a dose of 5 mg/kg, which is comparable to the control treatment. A comparable action was achieved with indomethacin compared to the control treatment [72].

#### 5.3.3. Cardiovascular Effects

*C. annuum* contains an anticoagulant that helps avert the clotting of the blood, resulting in strokes [4]. Natural α-amylase and α-glucosidase inhibitors from food-grade plants propose interesting approaches for the management of type-2 diabetes. The concomitantly related macrovascular complication of hypertension can be achieved by comparable extracts via the inhibition of the angiotensin I-converting enzyme (ACE). Nine types of pepper (*C. annuum*) were examined for their potent inhibitory activity against α-amylase, α-glucosidase, and ACE inhibitors. Numerous pepper extracts revealed a raised α-glucosidase inhibitory activity, not connected to the total phenolic content and the antiradical activity. Choice extracts, for instance, green pepper and long hot pepper, displayed a low or no inhibitory effect against α-amylase. Numerous aqueous extracts of red pepper displayed the uppermost ACE inhibitory activity [119]. An in vitro thrombolytic model was employed to observe the clot lysis property of *C. frutescens.* However, an in vitro thrombolytic model, *C. frutescens*, and a mixture of honey and *C. frutescens* revealed 57.40% and 44.54% clot lysis effects, respectively [120]. A recent study analyzed pepper oleoresin effects on gerbil males, and the researchers noticed that oleoresin reduced the triglycerides and cholesterol levels in the blood by 66% and 70%, respectively. It was hypothesized that this effect was due to a decreased intestinal absorption of exogenous cholesterol as a result of the increased biliary excretion of endogenous cholesterol after feeding some rabbits with a diet rich in cholesterol (1%) and chili pepper (1%) for 12 weeks [121]. As a result, the hematic concentrations of cholesterol, low-density lipoproteins (LDL) and very low-density lipoproteins (VLDL) were lower, and the risks of atheroma formation were reduced. It was also known that chili seeds contain lecithin, which is a natural emulsifier of fats that keeps the cholesterol in suspension in the blood, preventing it from settling on the arteries. Several studies have indicated that capsaicinoids inhibit platelet aggregation. Marbut et al. [46] confirmed this characteristic of capsaicin [122]. The ethanolic extracts of *C. annum* efficiently regulate arthritis growth. The arthritis scores of adjuvant induced arthritis (AIA) were considerably reduced with the leaf extracts of *C. annum* [123]. The nanovesicle formulation displayed a healthier acceptability and recognition in human and animal models [124]. A paste of leaves is used in the management of arthritis [125]. Moreover, recent research documented the effect of capsaicinoid on the plasma fat and aorta functionality, including fecal sterol excretion; biomarkers of cholesterol absorption; atherosclerotic plaque development; and the gene expression of major enzymes, transporters, and receptors involved in cholesterol metabolism in hamsters [126]. Srinivasan et al. [127] revealed the efficacy of capsaicinoids in decreasing the plasma total cholesterol, enhancing the lipoprotein profile, and reducing the aortic plaque in high-cholesterol-fed conditions. They revealed that capsaicinoids lowered the plasma campesterol/cholesterol ratio, indicating a decrease in cholesterol absorption. Additionally, capsaicin caused apnea, decreased heart rate, and produced hypotension when injected intravenously or into the carotid sinus [44]. The cardiovascular system comprises capsaicin-sensitive sensory nerves which participate in the control of the cardiovascular role via the activation of transient potential vanilloid 1 (TRPV) and substance P and the release of calcitonin gene-related peptide (CGRP). Several studies have indicated that capsaicinoids have therapeutic roles in curing disorders of the cardiovascular system [128,129,130]. Capsaicin also prevented platelet aggregation and the activity of clotting factors VIII and IX, a property which decreases the occurrence of cardiovascular diseases [128,129].

#### 5.3.4. Anti-Obesity Effect

The anti-obesity property of the aqueous extracts of seven edible green peppers belonging to *C. annuum* L. of the Solanacese family—Putgochu (Pca), Oyee gochu (Oca), Kwari putgochu (Kca), Green pepper (Gca), Yellow paprika (Yca), Red paprika (Rca), and Cheongyang gochu (Cca)—was investigated via the assessment of the lipoprotein lipase (LPL) mRNA expression level in 3T3-L1 cells (mouse pre-adipocytes) [119]. After capsaicin removal by chloroform defatting, the freeze-dried powder of Cca was administered to 3T3-L1 cells, and the anti-obesity effects were observed by assessing the LPL mRNA level via the RT-PCR protocol. Of the seven diverse *C. annuum* varieties, a substantial reduction in the LPL mRNA expression level of 50.9% in the Cca treatment was observed compared to the negative group [130]. The chronic administration of capsiate, a non-pungent capsaicin-related compound derived from a highly concentrated CH19 sweet pepper fruit, was reported to enhance the loss of fat in both humans and rodents without any toxic effect. This anti-obesity activity was induced by accelerating the oxidation of basal fatty acids in the mitochondria [131,132]. In particular, the oral administration of capsiate has been shown to stimulate the transient potential vanilloid 1 (TRPV1) receptor, which in turn raises the lipolysis of fat tissues through the sympathetic nervous system and thermogenesis [132]. Moreover, Snitker et al. [55] stated that chronic capsiate administration increases O_2_ consumption, enhancing energy expenditure and fat oxidation and reducing abdominal adiposity in experimental animals and in humans, especially those with a high BMI. Thus, capsiate could represent a promising approach in antiobesity programs, especially for individuals during a time of limited energy intake [133]. Ohyama et al. [134] revealed the synergistic antiobesity effect of capsiate, dihydrocapsiate, and nordihydrocapsiate, and cold temperature combination by stimulating beige adipocyte biogenesis and energy expenditure.

#### 5.3.5. Immunosuppressant and Memory Enhancing Activity

The direct administration of *Capsicum* extract and capsaicin caused the downregulation of interleukin (IL)-2, interferon (IFN)-gamma, and IL-4 and IL-5 production. Furthermore, a flow cytometric analysis showed a reduced population of CD3 (+) cells and an increase in CD19 (+) cells. The immunological effects of red pepper (*C. annuum* L.) extracts and its chief pungent capsaicin was evaluated on T helper 1 (Th1) and 2 (Th2) cytokine generation in cultured murine Peyer’s patch (PP) cells in vitro and ex vivo. In ex vivo research using PP cells detached from mice after oral treatment with *Capsicum* extract (10 mg/kg/day) for 4 successive days, IL-2, IFN-gamma, and IL-5 were found to upsurge in reaction to concanavalin A (Con A). Treatment with 3 mg/kg/day of capsaicin also improved the IL-2, INF-gamma, and IL-4 generation in reaction to Con A activation but did not sway the production of IL-5 [135]. Green chili is a favorable memory enhancer, and the fundamental mode of action of green chili seems reliant on (i) the enhancement of memory in exteroceptive models, (ii) the retrieval of memory shortfalls, (iii) the improved scavenging of radicals, and (iv) the inhibition of acetylcholinesterase.

#### 5.3.6. Antiangiogenic and Anti-Neoplastic Effects

Angiogenesis refers to the growth of new blood vessels from pre-existing vasculature. It is a complex multistep process involving endothelial cell activation, cell proliferation, invasion, chemotactic migration, and differentiation into new blood vessels [136]. The acquisition of an angiogenic phenotype is considered to be a vital step in tumor progression [137,138]. Several congruent studies indicate that the transition from an in situ carcinoma to invasive cancer (for solid tumors) must be accompanied by neovascularization. Therefore, the suppression of angiogenesis is a highly effective strategy for the treatment of multiple cancers [139,140,141], and one for which capsaicinoids may have relevant therapeutic potential, as they demonstrate significant anti-angiogenic activity in both cell culture and mice models. The effects of capsaicin on angiogenesis were investigated using multiple models [142]. Capsaicin has been proven to inhibit vascular endothelial growth factor (VEGF)-induced angiogenesis in ex vivo rat aortic rings models, Matrigel model systems, in vivo Matrigel plug experiments, and chicken chorioallantoic membrane (CAM) models [142]. Capsaicin robustly inhibited VEGF-induced endothelial cell proliferation and invasion [142]. Similarly, the non-pungent capsinoids, capsiate and dihydrocapsiate, inhibited VEGF-induced angiogenesis in both cell culture and mouse models [143]. Capsiate and dihydrocapsiate inhibited the VEGF-induced endothelial permeability and formation of cell-cell junctions by the direct inhibition of Src kinase activity and the phosphorylation of its downstream substrates (e.g., vascular endothelial cadherin and p125^FAK^). The anti-angiogenic activity of capsiate and dihydrocapsiate was found to be independent of the TRPV1 receptor [143]. The antiangiogenic activities of capsiate and dihydrocapsiate were compared to those shown by capsaicin. These non-pungent capsaicinoids (capsiate and its associated compounds) may be more important than capsaicin in the treatment of cancer; however, there are no records of other natural and synthetic capsaicin analogs having antiangiogenic action [144].

There are contradictory reports on the anti-cancer activity of capsaicinoids. The anticancer property of capsaicin was documented in diverse kinds of cancer cell; capsaicin blocked breast cancer cell migration and killed prostate cancer cells. Dihydrocapsaicin induced autophagy in HCT116 human colon cancer cells. Capsaicin also inhibited the growth of leukemic cells. The investigation of the mechanism of action of capsaicin has shown that it induced cell cycle arrest and apoptosis and inhibited cellular metabolism. Capsaicin selectively thwarted the growth and induced the apoptosis of cancerous cell lines. However, the biochemical constituents of capsaicin (such as the reactive phenoxy radicals) might assault the DNA and activate mutagenicity and cancerous transformation [145]. Many studies have shown that low doses of capsaicin inhibit the development of many forms of human cancers. Capsaicin has a deep anticarcinomic effect on prostate cancer cells, causing the cell death of both androgen receptor-positive and negative prostate cancer cell lines linked to the elevation of the antibodies p53, p21, and Bax [146]. Besides this, capsaicin efficiently inhibited tumor growth and induced apoptosis with no toxic effects [147]. All capsaicin-like compounds (natural capsaicinoids or synthetic capsaicin mimetics) have displayed growth-inhibitory effects against several cell lines [148]. For instance, capsanthin, capsanthin 3′-ester, capsanthindiester, capsorubin, capsorubindiester, capsanthin 3,6-epoxide, cucurbitaxanthin A-3′ester, and β-carotene isolated from the fruits of *C. annuum* validated potent in vitro anti-tumor-promoting activity, with inhibitory effects on the Epstein–Barr virus early antigen activation induced by the tumor promoter 12-*O*-tetradecanoyl-phorbol-13-acetate [149]. After the incubation of adenocarcinoma cell lines with capsaicin for 24 h, the cell viability was reduced considerably in a concentration-dependent manner, and the apoptotic bodies significantly increased [150]. Additionally, nordihydrocapsiate exhibited a potent chemopreventive effect in an in vivo two-stage model of mouse skin carcinogenesis. These results may indicate that nordihydrocapsiate can provide defense against skin cancer and shows a better proapoptotic activity than capsaicin in Jurkat cells [144]. These capsiates act by inhibiting the transcription factor nuclear factor *κ*B, increasing reactive oxygen species, and causing the loss of mitochondrial membrane potential [151]. N-acylvanillamides (N-AVAMs), synthetic non-pungent capsaicin analogs, are among the most common compounds studied for their anticancer and analgesic activity. Several studies have revealed their anticancer activity against different human cancer cell lines [152,153]. Recently, the growth-inhibitory effect of unsaturated N-AVAMs (UN-AVAMs) has been investigated. For instance, Tuoya et al. [154] reported that dohevanil, a UN-AVAM, induced higher apoptosis than capsaicin against MCF-7 human breast cancer cells in vitro. Moreover, several UN-AVAM compounds showing different affinities for human TRPV1 receptor have been synthesized by Appendino et al. [155]. The combinatorial anticancer effect of capsaicin analogs with traditional chemotherapy or radiation is an interesting field of study and research. As well, the production of non-pungent second-generation capsaicin mimetics with antiangiogenic and anticancer activities will open the way for new therapeutic interventions for human cancers [144].

#### 5.3.7. Anti-Invasive and Anti-Migratory Activities of Capsaicinoids

A critical step in the metastatic process is the migration and invasion of cancer cells into the surrounding blood vessels, lymph nodes, and stroma [156]. There are contradictory studies about the effect of capsaicin on tumor cell migration and invasion. Additionally, Yang et al. [157] revealed that capsaicin induced human colon carcinoma cell invasion through signal transducer and activator of transcription 3 (STAT-3) and Akt/mTOR-dependent pathways. Other reports have documented that capsaicin exhibits anti-invasive and anti-migratory activities against thyroid cancer, breast cancer, bladder cancer, small cell lung cancer (SCLC), cholangiocarcinoma, and melanoma [158,159]. Capsaicin’s anti-migratory and anti-invasive activity in thyroid cancer has been shown to be induced by a TRPV1-dependent mechanism [158]. Capsaicin has been found to enhance the invasion of TRPV1-null 5637 human bladder cancer cells [160]. Capsaicin’s pro-invasive activity correlates with increased granzyme A (GZMA) and insulin growth factor production (IGF-1), and matrix metalloproteinase-9 (MMP-9) activation in TRPV-null cells. Capsaicin utilizes several signaling pathways to control cancer cell migration and invasion, including the MMP signaling pathway; adenosine monophosphate (AMP) activated protein kinase (AMPK); epithelial–mesenchymal transition (EMT) activation; VEGF; intracellular calcium elevation; tumor-associated NADH oxidase (tNOX); MMPs; Akt; Wnt-Hedgehog regulation; and p38 MAP kinase, extracellular signal-regulated kinase (ERK), epidermal growth factor receptor (EGFR), AP-1, NF-kB, and Rac1 inhibition [161,162]. The capsaicin analogs, arvanil and olvanil, demonstrated higher anti-invasive activity against a panel of human SCLC cell lines relative to capsaicin, and this activity was independent on TRPV1 and regulated by the AMPK pathway activation [159]. The anti-invasive activity of capsazepine, another capsaicin analog, against human prostate cancer cells was investigated by Lee et al. [163]. They found that capsazepine strongly inhibited DU145 human prostate cancer cell invasion by inhibiting the Janus kinase (JAK)/STAT3 pathway.

#### 5.3.8. Anti-Metastatic Activity of Capsaicinoids

Several studies have investigated the sensory effects of capsaicin on tumor metastasis [164]. For example, O’Neill et al. [165] as well as Erin et al. [166] revealed that high concentrations of capsaicin led to sensory neuron deactivation. Yang et al. [157] also demonstrated capsaicin’s effect on the metastasis of CT26 murine colorectal carcinoma cells. Venier et al. [167] examined capsaicin’s anti-metastatic activity in a transgenic adenocarcinoma of the mouse prostate (TRAMP) model of prostate cancer. They revealed that capsaicin administration greatly decreased the metastatic burden in the model TRAMP mice. This research indicates that capsaicin may have a strong anti-metastatic activity [167].

#### 5.3.9. Analgesic and Antiplatelet Effects

The analgesic effects of capsaicin are significantly improved through inflammation, supporting the fact that the activation of vanilloid receptor type 1 could possibly constitute an appropriate approach to thwart inflammatory hyperalgesia [168]. The neuropeptide substance P has been involved in the pathogenicity of inflammation and pain in arthritis. A significant pain reduction of 57% and 33% was documented by a 0.025% capsaicin cream in treating osteoarthritis and rheumatoid arthritis in patients, respectively [169]. It was indicated that the management of individuals with headaches with capsaicin may make inactive sensory neurons by decreasing the nerve terminals of the substance P, which indicates that intranasal capsaicin offers a different management option for the handling of this disease [170]. The analgesic effect of SDZ 249–665, a capsaicin analogue, has been reported in a nociceptive pain mouse model showing an anti-hyperalgesic effect in rat and guinea pig inflammatory pain models. It was more effective than capsaicin by 45 times when administered orally and 3 times subcutaneously. The absence of the excitatory and pungent effects associated with capsaicin constitutes a significant possible benefit of SDZ 249-665 [171]. The analgesic activity of UN-AVAMs compounds has been completely restored by introducing long-chain unsaturated fatty acids. Structural activity experiments tested the unsaturation degree in these side chains, and the side chain length generates analogs of capsaicin with an enhanced analgesic activity and binding profile to TRPV1 [153]. Capsaicin was discovered as a strong inhibitor of platelet aggregation and release reaction. It reduced the hemolysis of red blood cells (RBCs) caused by hydrogen peroxide. Capsaicin has a membrane steadying effect by the interference of the stimulation of the phospholipase A2 [172].

#### 5.3.10. Diabetic Neuropathy and Gastroprotective Effects

The anti-diabetic effect of *C. annuum* has been demonstrated and takes place through various mechanisms, such as the inhibition of α-glucosidase, α-amylase, and antioxidant activities; the insulin mimetic, weight control, and hypolipidemic effects of this plant; and the activation of TRPV1, which leads to improved insulin resistance, the suppression of inflammation, and the control of glucose homeostasis. Moreover, *C. annuum* can act by enhancing the insulin sensitivity in peripheral tissues, stimulating the secretion of glucagon-like peptide-1 (GLP1), improving glucose tolerance, protecting β cells from apoptosis, and decreasing the level of fasting glucose/insulin and adipocytokine gene expression [173]. Sanati et al. [174] reported that short-term red pepper treatment decreased the level of serum glucose in diabetic rats, whereas a long-term treatment decreased the levels of triglycerides only. Moreover, Baek et al. [119] studied the inhibitory property of *C. annuum* against α-amylase, α-glucosidase, and angiotensin I-converting enzyme (ACE) inhibitors. Clinical trials carried out in individuals with diabetic neuropathy established a 50% enhancement in pain status with the usage of capsaicin for 22 weeks. It acts by exciting nociceptive C-afferent neurons, triggering the release of the substance P, which is important in order for the transmission of nociception to happen in the nervous system. The repeated application of capsaicin reduces substance P, resulting in pain sensation inhibition [80]. The crude extract of the fruit was discovered to thwart glucose absorption, which is partly accountable for reducing blood glucose [175]. The regular ingesting of chili may mitigate postprandial hyperinsulinemia [176]. Capsaicin reduced the gastric basal output and improved the non-parietal constituent of gastric secretory responses, gastric draining, and the release of glucagon. Capsaicin stopped indomethacin- and ethanol-induced gastric mucosal injury; meanwhile, capsaicin itself improved the gastric transmucosal potential difference. It can be summarized that capsaicin represents a new orally applicable gastro-protective agent in individuals with diverse chemical and *Helicobacter pylori*-induced mucosal damage and in numerous ailments necessitating treatment with non-steroidal anti-inflammatory drugs [177].

#### 5.3.11. Antiulcer and Respiratory Properties

*C. frutescens* was used as a curative against severe ulcers induced by aspirin at dosages of 300 and 600 mg/kg, and the outcome showed that the administration of the extract decreases the amount of gastric juice, reduced the length of gastric ulcers, and improved the histology alterations [178]. Red pepper has been employed for a long time as a digestive. Traditionally, conventional ulcer medicines avoid pepper, whereas herbalists recommended it. A study indicated that capsaicin inhibits in the vitro development of *Helicobacter pylori* at 10 µg/mL, negatively affecting the genesis of peptic and duodenal ulcers. In addition, capsaicin stimulates the formation of prostaglandins [179] by nerves sensitive to capsaicin. This research showed that the intake of 0.1 and 0.5 mg/kg of capsaicin after administration with indomethacin (NSAIDs) reduces the possible gastric lesions consequential to the use of NSAID agents. These lesions are normally caused by the NSAID-mediated reduction in prostaglandin production by the gastric mucosa. In fact, prostaglandins prevent gastric lesions because they act as stimulants of the production of mucus and bicarbonate [122]. Cough sensitivity to capsaicin is utilized as a device in biomedical studies. The cough sensitivity to capsaicin is used in the clinical evaluation of cough suppressants, such as benzonatate and guaifenesin [180]. In biomedical research, capsaicin weakens the nasal mucosa and lessens the allergic signs of nasal reaction or pain caused by additional agents [181]. Capsaicin offers a defensive approach by bolstering the lung defense system. Capsaicin has realized extensive usage in biomedical practice, since it causes coughing in a concentration-dependent and replicate manner [182].

#### 5.3.12. Dermatological Conditions, Pruritus, Psoriasis, and Rhinitis

**Figure 2:**
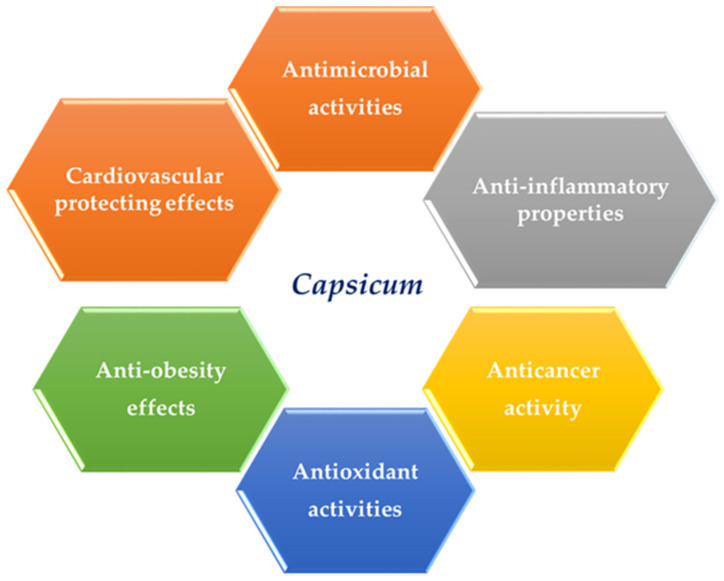
Summary of the biological activity of *Capsicum*.

Capsaicin, a recognized inhibitor of cutaneous vasodilatation, offers aid in reasonable and adverse psoriasis. A considerably enhanced decrease was perceived on parts administered with capsaicin comparable to the sides administered with buffer. The side effects were observed closely by the individuals during the early use of the examined medicine; however, they lessened or disappeared upon sustained usage [183]. Topical 0.075% capsaicin cream was positively utilized in the management of acute lipodermatosclerosis in pregnant woman [184]. Capsaicin principally influenced the slow steady curative of the skin lesions. Topical capsaicin is revealed to efficiently cure pruritus [185]. It was discovered that individuals with very itchy platelet-rich plasma (PRP) administered with capsaicin were seemingly discharged [186]. Capsaicin is also utilized in managing prurigo nodularis [187]. Topical capsaicin is revealed to efficiently cure pruritus, which is relevant to psoriasis [185,188], pityriasis rubra pilaris [186], psoralen-ultraviolet-light (PUVA) treatment [189], prurigo nodularis [190], and pruritus [187]. However, large, high-grade clinical trials are deficient. An evidence-based review of botanicals for dermatologic situations showed the data from two 6-week clinical trials (*n* = 241) on the usage of capsaicin cream on psoriasis. Capsaicin was better than the placebo for the respite of the signs of scaling, thickness, erythema, and pruritus. Burning at the application site was the most frequently documented severe effect [191]. A Cochrane review of the effectiveness of inhaled capsaicin in allergic rhinitis was discovered to lack proof to aid clinical use [188,189,190]. The biological activity is shown in Figure 2 and Table 6.

## 6. Dosing

For external uses, capsaicin and *Capsicum* creams are accessible in numerous ways, from capsaicin 0.025% to 0.075%, and are applied up to 3 to 5 times daily. *Capsicum* plasters comprising powdered *Capsicum* 345.8 mg and *Capsicum* 34.58 mg tincture per sheet (12.2 × 16.4 cm) have been assessed for postoperative pain and nausea [195]. A high-concentration dermal patch (8% w/w capsaicin) has been utilized in HIV-linked neuropathy [196] and intractable pain [197]. Low-strength (0.006%) capsaicin ointment was utilized in the atrial of itching in pruritus ani, with higher strengths leading to anal burning [187].

## 7. Pregnancy/Lactation

Studies in animals have displayed both positive and negative effects. Capsaicin crosses the placenta and was revealed to reduce substance P in the fetus with a neurotoxic effect. The growth rates of rat pups were lesser, and an abnormal testicular descent was observed in pups born to capsaicin-fed rats. However, no differences in rat pup malformations, epididymal or testicular weight, or plasma progesterone were established in other studies [198].

## 8. Toxicity of Pepper

Though most studies reveal that pepper and its bioactive compounds are safe, studies have specified its linked to cancer risks. Hwang et al. [199] stated that capsaicin might be associated with skin cancer. High gallbladder cancer (GBC) occurrence rates discovered were associated with high red chili pepper ingestion [200,201]. Szallasi et al. [202] reviewed the mutagenic effects of capsaicin on bacteria and mammals, and discovered contradictory outcomes. Although they resolved that capsaicin is not toxic (not mutagenic or weak mutagenic), the outcomes of tests displayed that ingestion might have carcinogenic or co-carcinogenic effects [202]. Within the body system, this may result in gastrointestinal cramping, discomfort, and diarrhea. Topically, this might trigger painful irritation of the mucous membrane [203]. The oral LD_50_ values for capsaicin are 161.2 mg/kg (rats) and 118.8 mg/kg (mice), with the hemorrhage of the gastric fundus detected in some of the animals that died. Although capsaicin is thought as safe and efficient as an external analgesic counterirritant, high doses administered over a protracted period of time can produce chronic gastritis, renal injury, hepatic damage, and neurotoxic effects [204,205]. Saito and Yamamoto [201] discovered that the acute oral LD_50_ values of capsaicin were estimated to be 97.4 and 118.8 mg/kg in female and male mice, respectively, and 148.1 and 161.2 mg/kg in female and male rats, respectively. The animals either died within 26 min of dosing, or revealed no further signs 24 h after dosing [206]. Acute myocardial infarction was described in a 40-year-old man admitted to a emergency division with complains of chest discomfort and dyspnea after exposure to pepper gas that spurted into the surroundings during a social event [184]. *C. annuum* must not be utilized during pregnancy and lactation in individuals with hypersensitivity and in children. The plant must not be employed on open wounds or abrasions, or near the eyes [207]. It also reduced the actions of *α*-adrenergic blockers, clonidine, and methyldopa [98]. Epidemiological and case-control studies have revealed a 2- to 3-fold upsurge risk of cancers, as well as oral, pharyngeal, and esophageal studies, and a movement toward an augmented risk of gall bladder, stomach, and colon cancers with chili consumption. There are varied results for capsaicin as a carcinogen, cocarcinogen, and anticarcinogen [198]. The preliminary interaction of capsaicin with the skin offers a fierce irritation with successive desensitization. In protracted exposure and in adverse cases, tenacious dermatitis with adverse effects may occur [208]. The interaction of the eyeball with *Capsicum* produces redness, inflammation, and tearing. More adverse indications comprise tenacious pain and foreign body sensation [209]. Reduced tear production and damaged corneal reflex lacrimation have been documented [210]. The toxic effects of *Capsicum* on the eyeball were documented by numerous researchers [208,211]. Contact may happen via breathing, producing an immediate redness of the mucous membranes [212]. The other signs documented comprise adverse coughing, mucus secretion, difficulty in breathing, and chest tightness [213]. Internally, *Capsicum* may produce gastrointestinal constrain, discomfort, and diarrhea. It produces sore irritation of the skin. A high dosage of capsaicin over a protracted time can produce renal injury, hepatic impairment, and neurotoxic effects [204]. Some studies have described systemic signs, comprising hyperventilation and lung edema. The severe upsurge in blood pressure might produce pain and amplify strokes [213]. A summary of the toxic effects of *Capsicum* is shown in Table 7.

## 9. Adverse Reactions

Reactions to bananas, kiwi, chestnut, or avocado may dispose individuals to *Capsicum* (pepper) allergy [214]. Allergic reactions to paprika occur in patients with “mugwort-celeryspice” syndrome [215].

## 10. Drug Interaction

Interactions were documented with the concurrent treatment of *C. annuum* with aspirin and salicylic compounds [216].

## 11. Pharmacokinetics of Capsicum and its Related Compound (Capsaicin)

Chaiyata et al. [217] investigated the metabolic rate (MR) of 5 g of fresh chili pepper in Thai women. They revealed that the MR immediately increased after fresh chili pepper ingestion and lasted for up to 30 min. Moreover, Weerapan Khovidhunkit [218] showed that the oral administration of 5 g gel capsules of *Capsicum* in humans maintained insulin levels, and that capsaicin can be first detected in the plasma at 10 min with a peak plasma concentration (C_max_), T_max_, AUC _(0-t)_, and T_1/2_ of 2.47 +/− 0.13 ng/mL, 47.08 +/− 1.99 min, 103.6 +/− 11.3 ng × min/mL, and 24.87 +/− 4.97 min, respectively. Early studies have reported the liver metabolism of capsaicin [219]. Several research laboratories have examined the liver metabolism of capsaicinoids in vitro via liver microsomes [220]. The metabolism was quicker in the rat and human hepatic microsomes than the corresponding 9000 g supernatant. Chanda et al. [221] perceived that capsaicin was totally metabolized within 20 min in rat and human microsomes. The highest levels were detected in diverse tissues at several times. The blood and intestines revealed a maximum concentration at 1 h, hepatic at 3 h, and renal at 6 h [222]. The absorption of orally administered capsaicin seems to be quick relative to the other two spice principles, as specified by its maximum concentration in the intestinal tissue at 1 h [222]. About 6.3 percent of the administered capsaicin was expelled as such in the feces over a period of 4 days, with the peak excretion happening on the first day of oral intake. Therefore, approximately 94 percent of orally administered capsaicin is digested. Only a small part of capsaicin was also expelled intact in urine (0.095%) [223]. Kawada et al. [224] have documented from an in vivo study in rats that the gastrointestinal absorption of capsaicin is rapid, and about 85 percent of the dose was absorbed within 3 h. Additionally, the absorbed capsaicin is willingly transported to the portal blood (about 85%) and partially metabolized during absorption to 8-methyl nonanoic acid. The metabolism of capsaicin was discovered to be comparable in human, rat, and dog microsomes [225]. In the tissue distribution and excretion of orally administered piperine, a maximum of 10.8 percent of administered piperine was observed in these tissues by 6 h after administration [222]. The quantity of piperine in the serum was 6.07, 9.75, and 11.1 µg/mL at 1, 3, and 6 h, respectively, which weakened significantly to 0.93 µg/mL at the end of 24 h and was nil in the blood after 4 days. Piperine was not noticeable in the urine at any time interval. Alternatively, 3.64 percent of the administered piperine was excreted as such in the feces over a period of 4 days, with the peak excretion occurring on the first day of oral intake. Therefore, the absorption of the administered piperine was about 96 percent [222]. Three key metabolites recognized for capsaicin were 16-hydroxycapsaicin, 17-hydroxycapsaicin, and 16 and 17- dihydrocapsaicin. This was mostly removed by the kidneys, with a small amount defecated in the feces and urine [99,226]. Capsaicin is well absorbed from the topical application on the skin, when a 3% capsaicin solution applied topically was speedily absorbed and reached optimum concentration. The shelf-life of capsaicin was roughly 24 h [99].

## 12. Future Directions

A number of unpleasant side effects are associated with the pungent nature of capsaicin after oral administration which hinder its use as a possible therapeutic agent. Such adverse effects, including sweating, eye tearing, gastric pain, and ulcers, as well as its short half-life and low bioavailability, mostly promote patients to stop its use, making any clinical trials useless [203,209]. Several approaches were identified for enhancing capsaicin’s bioavailability, including iontophoresis, hydrogel formations, and encapsulation in liposomes. Nanotechnology has recently been used to generate continuous release formulations of capsaicin [227,228]. For instance, some capsaicin formulations can induce a more persistent and sustained release of capsaicin to the site of the target organ. Tan et al. [229] revealed that most of the sustained-release topical capsaicin formulations exhibit greater skin permeability than capsaicin creams. Other capsaicin formulations exhibit a reservoir effect by reducing the capsaicin dose needed for treatment, thus resulting in less side effects, such as capsaicin formulations based on nanoparticles, leading to minimum discomfort in animal models. Capsaicin nanoparticles can also be used in combination therapy with other medications [230]. The discovery of second-generation capsaicin mimetics is another approach for resolving capsaicin side effects that would have a higher pharmacological activity than capsaicin [148]. Several natural capsaicinoids have been extracted from peppers and other natural sources. Likewise, synthetic capsaicin analogs were developed to manipulate the capsaicin pharmacophore. Long-acting analogs of capsaicin and capsaicin mimetics may form the basis of modern pharmacological treatments against a broad variety of human diseases, such as cancer, chronic pain, and atherosclerosis [230].

## 13. Conclusions

Pepper (*Capsicum* spp.) is among the oldest cultivated and most employed crops. Its usage started quite a long time ago and was considered to have its source in America. The genus *Capsicum* contains over two hundred species, with the fruits differing extensively in form, taste, and olfactory heat. *Capsicum* fruits are rich in capsaicinoids, carotenoids, flavonoids, vitamins, and minerals. The composition and quantity of these compounds vary according to the genotype and other factors. Capsaicin, the main active constituent isolated from *Capsicum* spp., is metabolized in the liver to three main metabolites—namely, 16-hydroxycapsaicin, 17-hydroxycapsaicin, and 16 and 17- dihydrocapsaicin—that were mostly removed by the kidneys, with a small amount defecated in the feces and urine. *Capsicum* produces redness, inflammation, tearing, tenacious pain, abdominal and skin irritation, coughing, and foreign body sensation. Capsaicin and *Capsicum* creams have been used externally and are accessible in numerous ways, from capsaicin 0.025% to 0.075%, and are applied up to 3 to 5 times daily. *Capsicum* plasters comprising powdered *Capsicum* 345.8 mg and *Capsicum* 34.58 mg tincture per sheet (12.2 × 16.4 cm) have been assessed for postoperative pain and nausea.

## Figures and Tables

**Table 1:** Nutritional and biochemical constituents of *C. annuum* fine particles at diverse harvest periods.

Nutritional and Biochemical Constituents	Value
Carbohydrate	55.33 ± 4.8–55.96 ± 3.3
Protein	20.19 ± 2.6–21.50 ± 4.5
Lipid	7.55 ± 3.9–9.75 ± 3.3
Dietary fiber	35.05 ± 1.4–37.07 ± 3
Total sugar	6.88 ± 0.47–11.19 ± 0.11
Energy content	1449.55–1573.17
Vitamin C	1360.2 ± 14.3–2020 ± 32.3
Total capsaicinoids	24.8 ± 5.5–59.7 ± 6.2
Scoville heat value	73–938
Potassium	2168 ± 147–2523 ± 280
Phosphorus	363 ± 43–453 ± 30
Magnesium	130 ± 15–146 ± 49
Calcium	41.62 ± 10–186 ± 54
Iron	31 ± 10–53 ± 15
Sodium	30 ± 4–71 ± 9;
Copper	1.06 ± 0.5–1.31 ± 0.3
Zinc	1.67 ± 0.2–2 ± 0.2

**Table 2:** The mineral constituents of oils extracted from two varieties of *C. annuum* (sweet and bell pepper).

Sweet Pepper		Bell Pepper	
Mineral Constituent	Value	Mineral Constituent	Value
Sodium	40 ± 0.01	Sodium	6.10 ± 0.01
Calcium	8.00 ± 0.01	Calcium	6.00 ± 0.02
Magnesium	9.60 ± 0.01	Magnesium	6.00 ± 0.01
Potassium	43.70 ± 0.13	Potassium	49.10 ± 0.06
Zinc	0.01 ± 0.00	Zinc	0.02 ± 0.00
Iron	9.00 ± 0.01	Iron	17.10 ± 0.00
Cadmium	ND	Cadmium	ND
Nickel	ND	Nickel	ND
Copper	0.30 ±0.00	Copper	0.16 ± 0.00

ND: not determined.

**Table 3:** Chemical constituents of *n*-hexane *C. annuum* extracts.

Compounds	Compounds	Ref.
2-heptanal (*E*)	2-decenal (*E*)	[47,48,49]
(2*E*,4*E*) 2,4-decadienal	Cadienal	
2-undecenal	Tetradecane	
Methyl 9-oxononanoate	Hexadecane	
2,6,10,14-tetramethyl	Pentadecane	
Phenol	2,6-*bis*(1,1-dimethylethyl)-4-methyl	
Heptacosane	Farnesol	
Hexadecene	Tetradecanal	
Heptadecane	Myristic acid methyl ester	
9-octadecene (*E*)	1-pentadecene	
Undecane	Exadecane	
Oleic acid	Oleic acid methyl ester	
Pentadecanoic acid methyl ester	Pentadecanoic acid	
Neophytadiene	2-pentadecanone, 6,10,14-trimethyl	
2-decene, 7-methyl-	Palmitic acid, 14-methyl- methyl ester	
(*Z*) pentadecanoic acid	Palmitic acid ethyl ester	
Margaric acid methyl ester	5-octadecene (*E*)	
1-octadecadienoic acid methyl ester	Linolenic acid methyl ester	
Phytol, stearic acid methyl ester	Hexadecanamide	
Eicosane	Octadecanal	
Nonadecanoic acid methyl ester	1-octadecene	
Linoleic acid	Docosane	
Arachidic acid methyl ester	Octadecane	
9-octadecenamide (*Z*)-1-heneicosyl formate	Octadecanamide	
Heneicosanoic acid methyl ester	Cyclodocosane ethyl	
4-hexenoic acid, 3-methyl-2,6-dioxo-	Cyclotetracosane	
Behenic acid methyl ester	Palmitic acid 2-hydroxy-1-(hydroxymethyl) ethyl ester	
Pelargonic acid vanillylamide	Linoleic acid 2-hydroxy-1-(hydroxymethyl) ethyl ester	
Tricosanoic acid methyl ester	2-monolinolenin	
Stearic acid	2-hydroxy-1-(hydroxymethyl) ethyl ester	
Cholest-5-en-3-ol (3β)	Lignoceric acid methyl ester	
Ergost-5-en-3-ol, (3β,24*R*)-	Vitamin E	
Stigmast-5,22-dien-3-ol (3β,22*E*)	Stigmast-5-en-3-ol, (3β,24*S*)	
β-Amyrin	Viminalol	
Stigmast-4-en-3-one		

**Table 4:** The flavonoid and phenolic acid fraction of red pepper extract.

Compounds	Compounds	Ref.
*trans*-*p*-feruloyl-β-d-glucopyranoside;	*trans*-*p*-sinapoyl-β-d-glucopyranoside	[50,51,52,53]
Quercetin 3-*O*-α-l-rhamnopyranoside-7-*O*-β-d-glucopyranoside	Luteolin 6-*C*-β-d-glucopyranoside-8-*C*-α-l-arabinopyranoside	
*trans*-*p*-feruloyl alcohol-4-*O*-[6-(2-methyl-3-hydroxypropionyl] glucopyranoside	Apigenin 6-*C*-β-d-glucopyranoside-8-*C*-α-l-arabinopyranoside;	
Lutoeolin 7-*O*-[2-(β-d-apiofuranosyl)-β-d-glucopyranoside]	Quercetin 3-*O*-α-l-rhamnopyranoside and luteolin-7-*O*-(2-apiofuranosyl-4-glucopyranosyl-6-malonyl) glucopyranoside.	
Quercetin-3-*O*-l-rhamnoside		

**Table 5:** International Union of Pure and Applied Chemistry (IUPAC) name, and the chemical formula of bioactive molecules isolated from *Capsicum*.

Compound	Synonyms	Class of Compound	IUPAC Name	Chemical Formula	Examples of Plants Parts as Source	Ref.
Piperine	1-Piperoylpiperidine	Piperine is a *N*-acylpiperidine that is piperidine substituted by a (1*E*,3*E*)-1-(1,3-benzodioxol-5-yl)-5-oxopenta-1,3-dien-5-yl group at the nitrogen atom.	(2*E*,4*E*)-5-(1,3-benzodioxol-5-yl)-1-piperidin-1-ylpenta-2,4-dien-1-one	C_17_H_19_NO_3_	Constituent of pepper spp. (Piperaceae).	[68]
Capsaicin	Capsaicin	Capsaicin is a capsaicinoid. It has a role as a non-narcotic analgesic, a voltage-gated sodium channel blocker, and a TRPV1 agonist.	(*E*)-*N*-[(4-hydroxy-3-methoxyphenyl)methyl]-8-methylnon-6-enamide	C_18_H_27_NO_3_	Capsaicin is identified as the primary pungent principle in *Capsicum* fruits.	[60]
Dihydrocapsaicin	6,7-Dihydrocapsaicin	Dihydrocapsaicin is a capsaicinoid and analog and congener of capsaicin in chili peppers (*Capsicum*). Like capsaicin, it is an irritant.	*N*-[(4-hydroxy-3-methoxyphenyl)methyl]-8-methylnonanamide	C_18_H_29_NO_3_	Dihydrocapsaicin is found in pepper (*C. annuum*).	[69]
Nordihydrocapsaicin	*N*-(4-hydroxy-3-methoxybenzyl)-7-methyloctanamide	Nordihydrocapsaicin is found in herbs and spices.	*N*-[(4-hydroxy-3-methoxyphenyl)methyl]-7-methyloctanamide	C_17_H_27_NO_3_	Nordihydrocapsaicin is isolated from the pungent principle of red pepper (*Capsicum annuum*).	[69]
Capsiate	(4-hydroxy-3-methoxyphenyl)methyl (*E*)-8-methylnon-6-enoate	Lipophilic alkaloid is an analogue of capsaicin.	(4-hydroxy-3-methoxyphenyl)methyl (6*E*)-8-methylnon-6-enoate	C_18_H_26_O_4_	Non-pungent capsaicin-related substances found in all tested variants of the *Capsicum* genus of plants.	[70]
Dihydrocapsiate	4-Hydroxy-3-methoxybenzyl 8-methylnonanoate	Lipophilic alkaloid is an analogue of capsaicin.	(4-hydroxy-3-methoxyphenyl)methyl 8-methylnonanoate	C_18_H_28_O_4_	Non-pungent capsaicin-related substances found in all tested variants of the *Capsicum* genus of plants.	[70]
Nordihydrocapsiate	4-hydroxy-3-methoxybenzyl 7- methyloctanoate	Lipophilic alkaloid is an analogue of capsaicin.	(4-hydroxy-3-methoxyphenyl)methyl 7-methyloctanoate	C_17_H_26_O_4_	Non-pungent capsaicin-related substances found in all tested variants of the *Capsicum* genus of plants.	[70]
Capsiconiate	Coniferyl (*E*)-8-methyl-6-nonenoate	Secondary plant non-toxic alkaloid metabolites.	[(*E*)-3-(4-hydroxy-3-methoxyphenyl)prop-2-enyl] (*E*)-8-methylnon-6-enoate	C_20_H_28_O_4_	Non-pungent capsaicin-related substances found in all tested variants of the *Capsicum* genus of plants.	[71]
Dihydrocapsiconiate	Coniferyl 8-methylnonanoate	Secondary plant non-toxic alkaloid metabolites.	3-(4-hydroxy-3-methoxyphenyl)prop-2-en-1-yl 8-methylnonanoate	C_20_H_30_O_4_	Non-pungent capsaicin-related substances found in all tested variants of the *Capsicum* genus of plants.	[71]
beta-Carotene	Provitamin A	β- Carotene is a naturally-occurring retinol (vitamin A) precursor obtained from certain fruits and vegetables with potential antineoplastic and chemopreventive activities. As an anti-oxidant, beta carotene inhibits free-radical damage to DNA.	1,3,3-trimethyl-2-[(1*E*,3*E*,5*E*,7*E*,9*E*,11*E*,13*E*,15*E*,17*E*)-3,7,12,16-tetramethyl-18-(2,6,6-trimethylcyclohexen-1-yl)octadeca-1,3,5,7,9,11,13,15,17-nonaenyl]cyclohexene	C_40_H_56_	Fruits.	[72,73]
Rutin	Quercetin 3-rutinoside	Rutin is a flavonoid known to have a variety of biological activities, including antiallergic, anti-inflammatory, antiproliferative, and anticarcinogenic properties.	2-(3,4-dihydroxyphenyl)-5,7-dihydroxy-3-[(2*S*,3*R*,4*S*,5*S*,6*R*)-3,4,5-trihydroxy-6-[[(2*R*,3*R*,4*R*,5*R*,6*S*)-3,4,5-trihydroxy-6-methyloxan-2-yl]oxymethyl]oxan-2-yl]oxychromen-4-one	C_27_H_30_O_16_	Pepper fruit.	[74]
Quercetin	Xanthaurine	Quercetin is a polyphenolic flavonoid with potential chemopreventive activity.	2-(3,4-dihydroxyphenyl)-3,5,7-trihydroxychromen-4-one	C_15_H_10_O_7_	Pepper fruit.	[50]

**Table 6:** The biological activities of *Capsicum*.

Activities	Models	Extract	Bioactive Compound Mechanism of Action	References
Antimicrobial effects	*Micrococcus sp* (20 mm)*, Bacillus* (10 mm), *E. Coli* (17 mm)*, Pseudomonas sp* (16mm) and *Citrobacter sp* (15 mm)	The butanol extract of *Capsicum annuum* fruit	Capsaicin.	[88]
	*Salmonella typhimurium* and *Pseudomonas aeruginosa*	The ethanol extract (100 mg/mL) of *Capsicum annuum*.	Capsaicin.	[89]
	(*Staphylococcus aureus* UFPEDA02*, Enterococcus faecalis* ATCC6057, *Bacillus subtilis* UFPEDA 86*),* and Gram negative (*Escherichia coli* ATCC25922, *Klebsiella pneumonia* ATCC29665, *Pseudomonas aeruginosa* UFPEDA416) bacteria and one yeast strain (*Candida albicans* UFPEDA 1007)	*C. frutescens extract.*	CAY-1, a novel saponin obtained from *C. frutescens*, rupturing the membrane integrity of fungal cells.	[88,91]
	*Saccharomyces cerevisiae*, *Candida albicans*, *Candida parapsilosis*, *Candida tropicalis*, *Pichia membranifaciens*, *Kluyveromyces marxiannus* and *Candida guilliermondiiA. flavusA. niger*	*Capsicum frutescens* extracts.	Peptides obtained from chili pepper seeds.	[94] [93]
Antiviral	Guinea pigs against cutaneous herpes simplex virus	*Capsicum* extract.	Capsaicin, disrupting standard virus-neuron connections.	[192]
Insecticidal, anthelmintic and Larvicidal effects	*A. aegypti* mosquitoes	*Capsicum frutescens* extracts.	Extremely repellent extracts.	[115]
	Cercaria of *Schistosoma mansoni*	*Capsicum annuum* leaves extracts.	Water-soluble unsaturated compounds from the oils or their hydrolysis products.	[100]
Antioxidant properties	Adult men and women	*Capsicum annuum.*	Antioxidant properties of capsaicinoids gave additional advantage in the management of cardiovascular diseases.	[108]
Anti-inflammatory properties	Rat hind paw inflammation induced by subplantar injections of fresh egg albumin (0.5 mL/kg)	Ethyl acetate *C. frutescens* extract	Carotenoids.	[117]
Cardiovascular effects	Anesthetized dogs iv injections	Capsaicin (10-300 μg/kg).	Temporary increase in the mean systemic blood pressure trailed by a continued fall.	[193]
Antithrombotic and Vasodilatory Properties	Gerbil males	Pepper oleoresin.	Decreasing intestinal absorption of exogenous cholesterol as a result of the increased biliary excretion of endogenous cholesterol.	[121]
	Feed rabbits for 12 weeks with a diet rich in cholesterol (1%) and chili pepper (1%),	Chili pepper (1%).	Discovered the anti-atherosclerotic effects of pepper. At the end of 12 weeks, a reduced activity of cholesterol ester transfer protein (CETP) was witnessed, in contrast to the control group (rabbits feeding without pepper).	[194]

**Table 7:** Toxic effects of *Capsicum*.

Toxic Activities	Bioactive Compound/Type of Extract	Mechanism of Action of Toxicity	Ref.
Hemorrhage of the gastric fundus	Capsaicin 161.2 mg/kg (rats) and 118.8 mg/kg (mice).	Triggers painful irritation of the mucous membrane.	[204,205]
Myocardial infarction	40-year-old man admitted to emergency division with complains of chest discomfort and dyspnea after exposure to pepper gas.	Irritation of the mucous membrane.	[184]
Tenacious dermatitis	Capsaicin	Irritation of the skin.	[208]
